# The Pocket Formulary and Synopsis of the British and Foreign Pharmacopœias, Comprising Standard and Approved Formulæ for the Preparations and Compounds Employed in Medical Practice

**Published:** 1852-01

**Authors:** 


ART. X.—*The Pocket Formulary and Synopsis of the British and Foreign Pharmacopœias,* comprising standard and approved formulæ for the preparations and compounds employed in Medical Practice. By HENRY BEASLEY, M. D. First American from the London edition. Lindsay & Blakiston. Phila. 1852. 12mo. pp. 443.

This appears to be a carefully prepared synopsis of the standard pharmacopœias, and the list of works consulted in compiling the Pocket Formulary embraces nearly every important work on Materia Medica and Therapeutics in our language, and many from the French. A wide range of periodical literature has also been collated, for the purpose of enriching the work. The arrangement is alphabetical, with sufficient reduplication of synonyms to make it easy to refer to an article under any of its common names. The matter is necessarily very concise but it is clear and precise, and reference is given to authority in all important cases. Under the head of preparations and compounds employed as counter-poisons, we find Smith's antidote for prussic acid, viz: carb. iron, precipitated in the stomach by giving first the dose of carb. potas, and subsequently that of sulphate of iron. Formula, 10 gr. F. sulph. in f℥j water, and add f℈i of tinct. muriate (sesquichloride) of iron. In another vial, dissolve ℈j of subcarbonate of potash in two of water. Give the latter as quickly as possible, followed by the former.—The solutions should be kept in readiness.

*Magnesia.*—Caustic magnesia is also recommended as an antidote for white arsenic (arsenious acid), with some precautions to prevent its being injured by over-heating. It is recommended to prepare it fresh by the wet way by dissolving mag. sulph. in 25 times its weight of water; of caustic potash half the weight of the salt is to be taken and dissolved in 20 times its weight of water;—mix the solutions, collect the precipitate on a linen cloth, press it slightly, and if wanted immediately use it without further washing, half or three quarters of an ounce to a pint of water diffused. This form of magnesia is also one of the best antidotes to *acid* poisoning. In this connection we may also mention, that the hydrated protosulphuret and persulphuret of iron are the most reliable exhibitions as antidotes for metallic poisons generally, viz: for the salts of mercury, copper, and lead. A mixture of the above magnesia with moist sulphurets of iron would form an excellent antidote to be always on hand for metallic poisoning *including* white arsenic.

Ioduretted iodid of potassium, and purified animal charcoal are also recommended as antidotes for the vegetable alkaloids, viz: opium, salts of morphia, hemlock, aconite, belladonna, strychnine, colchicum, &c., but not for digitalis.

The profession will find "Beasley's Pocket Formulary" a very valuable vade mecum.

